# Uncovering the Association Between Complete AZFc Microduplications and Spermatogenic Ability: The First Reported Series

**DOI:** 10.7759/cureus.51140

**Published:** 2023-12-26

**Authors:** Kian Asanad, Elena Greenfeld, Stephen W Scherer, Ryan Yuen, Christian R Marshall, Kirk Lo, Brendan Mullen, Susan Lau, Keith A Jarvi, Mary K Samplaski

**Affiliations:** 1 Institute of Urology, University of Southern California Keck School of Medicine, Los Agneles, USA; 2 Department of Pathology and Laboratory Medicine, Mount Sinai Hospital Joseph and Wolf Lebovic Health Complex, Toronto, CAN; 3 McLaughlin Center and Department of Molecular Genetics, Mount Sinai Hospital, Toronto, CAN; 4 Division of Urology, Department of Surgery, Mount Sinai Hospital, Toronto, CAN; 5 Institute of Urology, University of Southern California Keck School of Medicine, Los Angeles, USA

**Keywords:** semen, male fertility, microarray, y chromosome, azfc microduplication

## Abstract

Purpose

This article aims to report the first series of men with complete *AZFc* microduplications and their clinical and reproductive characteristics.

Methods

We sampled 3000 men who presented for reproductive urology evaluation from 2012-2020, of which 104 men underwent high-resolution Y-chromosome microarray testing, and five men were identified to have complete *AZFc* microduplications. Medical, surgical, and reproductive histories were obtained. Semen and hormonal parameters as well as response to fertility therapies were recorded.

Results

Five men were identified as having complete *AZFc* microduplications. The mean age was 33.75 years, representing 0.2% (5/3000) of men presenting for fertility investigation, 4.8% (5/104) of men undergoing microarray testing, and 21% (5/24) of men with *AZFc* abnormalities. Two of the men had prior undescended testicles and one had several autoimmune processes. The mean follicle-stimulating hormone (FSH) was 5.5 IU/L, luteinizing hormone (LH) 3.6 IU/L, and testosterone 14.56 nmol/L. One man was azoospermic, one man alternated between severe oligospermia and rare non-motile sperm, one had variable parameters, with one semen analysis demonstrating azoospermia and a second demonstrating a total motile sperm count (TMSC) of 4 ×10^6^, one man was persistently oligospermic with TMSCs ranging 3.96-12.6 ×10^6^, and one man initially had severe oligospermia, with a mean TMSC of 1.5 ×10^6^, which increased to 21.7 ×10^6^ after intervention (varicocele embolization, clomiphene citrate). This last man then fathered a spontaneous pregnancy.

Conclusion

*AZFc* complete microduplications are a rare cause of spermatogenic failure but not an uncommon form of *AZFc* abnormality. Clinically, they represent a heterogeneous group, having a variable reproductive potential. Cases should be managed on an individual basis.

## Introduction

The human Y chromosome is remarkable for its high level of structural variability [1], and deletions, duplications, and inversions are relatively common [2]. Some of these variants are selectively neutral, and others may result in spermatogenic failure [3]. The azoospermia factor (*AZF*) region, encodes for 21 genes, and its three subregions *AZFa*, *AZFb,* and *AZFc*, have been mapped to Yq [4]. The *AZFc* region is comprised of massive palindromic sequences and amplicons, making it one of the most unstable regions in the human genome [5] and particularly prone to deletions and duplications [6].

It is well established that a complete *AZFc* microdeletion is a common cause of male factor infertility [7]. Partial microdeletions within the *AZFc* region are relatively common and present at various frequencies in different Y haplogroups [8, 9]. While the complete *AZFc* microdeletion is well associated with spermatogenic failure, the role of partial *AZFc* microdeletions on spermatogenesis is still debated [10]. With advances in detection methodology, increasing cases of partial *AZFc* microdeletions and Yq microduplications, especially those involving the *AZFc* region, have started to emerge [11-15].

Our group recently reported a custom high-resolution Y-chromosome microarray [16], which has enabled us to identify a group of men with complete Y-chromosome microduplications. Our high-resolution Y-chromosome microarray interrogates thousands of different loci on the Y-chromosome, in order to detect known Y-chromosome microdeletions and uncover other copy number variations of the Y-chromosome. From our previous study using the Y-chromosome microarray, 0/148 fertile men had *AZFc* microduplications. It is a direct result of this new technology [17], allowing for more thorough detection of Y-chromosomal abnormalities, that we herein report the first series of infertile men with complete *AZFc* microduplications. This article was previously presented as a meeting abstract at the 2023 American Urological Association (AUA) Annual Meeting on April 29, 2023 [18]. 

## Materials and methods

We identified 3000 men who presented for a fertility evaluation at a single male-infertility specialty clinic from 2012 to 2020. We included men with severe oligospermia (5 million sperm/ml) or non-obstructive azoospermia who underwent Y-chromosome microdeletion testing. Men with obstructive azoospermia, azoospermia secondary to exogenous testosterone usage, Klinefelter syndrome, or other chromosomal abnormalities were excluded. A total of 104 men underwent Y-chromosome microdeletion testing. In order to identify men specifically with complete AZFc microduplications, the charts of all men having Y-chromosome targeted microarray testing were manually reviewed. Five men were identified as having complete AZFc microduplications. These men were assessed for demographics, medical, surgical, family, and reproductive history, semen and hormonal parameters, as well as response to therapies. The collection of data and the analysis of the data in this database were approved by the Research Ethics Board of the Mount Sinai Hospital (IRB #14-0342-E).

Y-chromosome microdeletion testing was performed using targeted microarray in peripheral blood, as recently described by our group [17]. Our custom array included 10,162 probes with an average probe spacing of 1.3kb within the *AZFs* and probe spacing of 2.5 kb across the euchromatic region of the Y-chromosome and included a comprehensive list of unique targets that included 111 gene copies and 107 unique sequence-tagged site (STS) markers in addition to the three *AZF* regions and the palindromes within them. All eight STS markers recommended for use by the European Academy of Andrology and the European Molecular Genetics Quality Network best practice guidelines for the polymerase chain reaction (PCR)-based test, and sY14 (SRY), ZFY, sY84, sY86, sY127, sY134, sY254, and sY255 were also targeted in this targeted microarray [17]. Gene copy number variation was determined by the log ratio of probe signal intensity against a DNA reference. This approach has shown to be a reliable high-resolution alternative to multiplex PCR for the discovery of pathogenic chromosome Y microdeletions in male infertility [17].

Blood and semen samples were collected at several laboratories, based on patient convenience. All andrology laboratories used validated methodologies and performed their own quality control procedures. Semen samples were collected at least 48 hours, but not more than 7 days, after the time of last ejaculation. Furthermore, Fischer’s exact test was used to compare the rate of complete *AZFc* microduplications in the infertile population versus fertile controls from our previous study [17].

## Results

**Figure 1:**
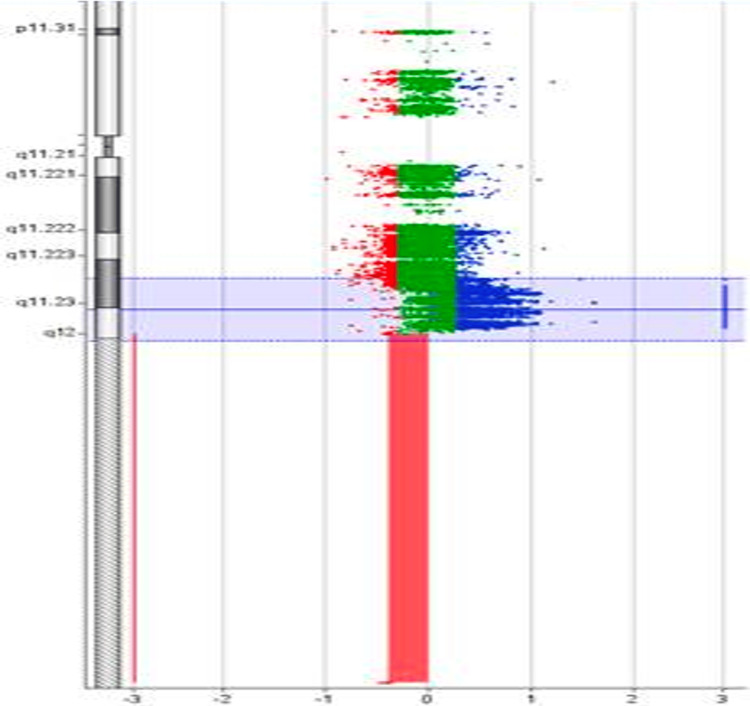
Graphical display of the Y-chromosome microarray demonstrating a complete AZFc microduplication seen in the blue highlighted section The green color represents a normal copy number. The blue color demonstrates a duplication or a gain of a copy number (shifted to the right). The red color demonstrates a deletion or a loss of a copy number (i.e. shifted to the left)

**Figure 2:**
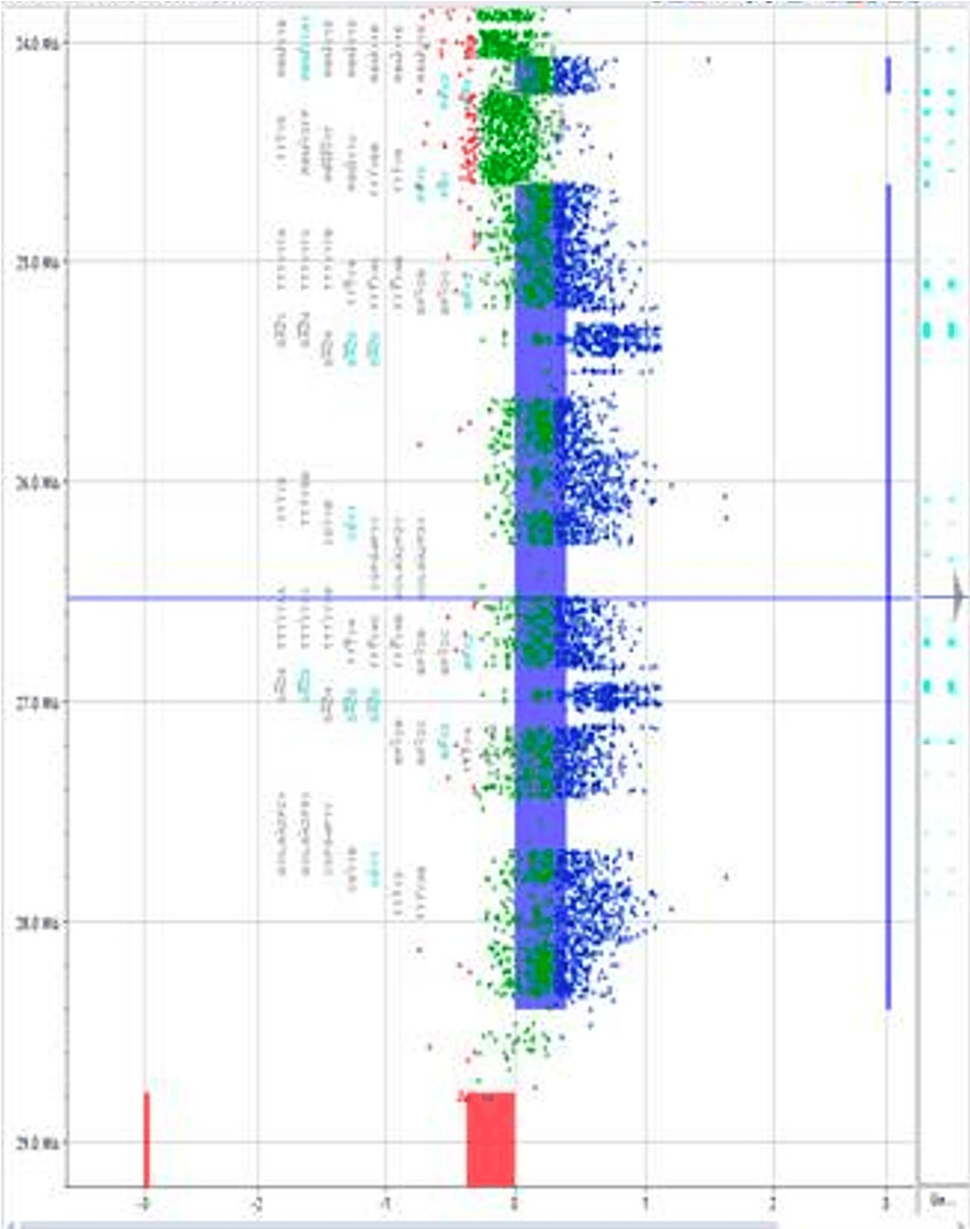
Magnified view of the selected AZFc region demonstrating a complete microduplication

From 2012 to 2020, 3000 men underwent new fertility evaluation, 104 underwent high-resolution Y-chromosome microarray testing, three had *AZFa* microdeletions detected, five had *AZFb* microdeletions detected, 19 had *AZFc* microdeletions detected, and five were identified as having complete *AZFc* microduplications. An example of our Y-chromosome microarray demonstrating an *AZFc* microduplication is shown in Figure 1 and Figure 2.

This represented 4.8% (5/104) of men undergoing Y-chromosome microarray testing, and 21% (5/24) of men with men with AZFc abnormalities. There were no *AZFa* and *AZFb* microduplications identified. There was a significantly higher proportion of *AZFc* microduplications in the infertile population (5/104) versus the fertile controls from our previous study (0/148) (p= 0.013). 

The clinical characteristics of these men are listed in Table 1.

**Table 1:** Cohort characteristics of men with complete AZFc microduplications DM = diabetes mellitus; TMSC = total motile sperm count; FSH = follicle stimulating hormone; LH = luteinizing hormone; T = testosterone; IVF = in vitro fertilization

Patient	Ethnicity	Age (years)	Medical History	Surgical History	Family History	Left testicular volume (mL)	Right testicular volume (mL)	Varicocele	TMSC (million)	FSH (IU/L)	LH (IU/L)	T (ng/dL)	Testis biopsy	Interventions	Pregnancy
																1	Indian	34	Right undescended testicle	Right inguinal hernia repair	None	20	12	None	Alternates between azoospermia and rare non-motile sperm	6	4	360	None	None	Planning for IVF with frozen sperm
2	Caucasian	33	None	None	None	18	18	None	Persistently azoospermic	11	4.2	375	Maturation arrest at the primary spermatocyte level	None	Planning for sperm retreival and IVF
3	Caucasian	33	Type 1 DM, asthma, autoimmune hepatitis, hypothyroid	None	None	16	16	None	First semen test = 4 million motile sperm Second semen test = azoospermic	7.8	3.4	527	None	None	1 male offspring after IVF
4	Caucasian	35	Undescended testicle, asthma	None	Father with delay in conception	18	22	Left side, Grade 2	First semen test = 1.8 million motile sperm Second semen test = 1.3 million motile sperm Third semen test = 21.7 million motile sperm (after intervention)	1	3	461	None	Varicocele embolization; Started on clomiphene citrate	Spontaneous pregnancy x2 after intervention; Couple has 2 boys, 2 years old and 1 month old
5	Middle Eastern	36	Psoriasis	None	Brother with oligospermia	20	20	None	First semen test = 8 million motile sperm Second semen test = 12.6 million motile sperm Third semen test = 3.96 million motile sperm Fourth semen test = 5.64 million motile sperm	1.7	3.4	375	None	None	Planning for IVF

The mean male partner age was 33.75 years (range 33-35 years). The mean female partner age was 30.5 years (range 27-33 years). All couples were seen for primary infertility, with a mean duration of infertility of 57 months (range 12-180 months). Two of the men reported that their fathers had impaired male factor fertility. One of these fathers required assisted reproductive technology for conception and underwent *AZFc* microarray testing at our clinic and was also found to have an *AZFc* complete microduplication. The other father reported that it took approximately four years to conceive the man that we were seeing in the clinic. Patient 5 reported that he had a brother who was also oligospermic, although Y chromosome microarray testing was not done since he lived on another continent. The man with a brother with subfertility was not one of the two men whose fathers had possible subfertility.

Patient 3 had a history of several autoimmune processes, including type 1 diabetes, asthma, autoimmune hepatitis, and hypothyroidism. Patient 1 and Patient 4 had a history of undescended testicles; one of them had it surgically fixed as an infant and the other's descended spontaneously. In both of these men, the testicle that had been undescended was smaller in size by 2-4 mL compared with the contralateral testicle. On examination, the mean testicular size was 18 cc (range 12-22 cc), consistent with other genetic spermatogenic insults. One man had a left-sided grade 2 varicocele.

The mean follicle-stimulating hormone (FSH) was 5.5 IU/L (range 1-11 IU/L). The mean luteinizing hormone (LH) was 3.6 IU/L (range 3-4.2 IU/L). The average endogenous testosterone (T) was 419 ng/dL (range 360-526 ng/dL). All men also had negative cystic fibrosis testing and normal karyotypes.

With respect to semen parameters, Patient 1 alternated between severe oligospermia and rare non-motile sperm. Patient 2 was persistently azoospermic on three semen analyses. Patient 3 had one semen analysis demonstrating azoospermia and a second demonstrating a total motile sperm count (TMSC) of 4 ×10^6^. Patient 4 had severe oligospermia with TMSCs of 1.8 and 1.3 ×10^6^. After an intervention, which included a varicocele embolization and clomiphene citrate therapy, his TMSC rose to 21.7 ×10^6^. Patient 5 was persistently oligospermic with TMSCs of 8, 12.6, 3.96, 5.64 ×10^6^.

Because of normal hormone levels, normal testicular volume, and persistent azoospermia, one man underwent a percutaneous testicular biopsy to rule out an obstructive process, which revealed maturation arrest at the primary spermatocyte level.

With respect to interventions, Patient 4 had a TMSC of 1.98 ×10^6^ at presentation (ejaculate volume 3 mL, sperm concentration 2 ×10^6^/mL, motility 33%). At repeat semen analysis his TMSC was 1.3 ×10^6^ (ejaculate volume 1 mL, sperm concentration 5.6 ×10^6^/mL, motility 24%). He was found to have a left grade 2 varicocele, with discrepant testicular volumes of 18 versus 22 cc. His FSH was 1 IU/L, LH 3 IU/L, and T 461 ng/dL. He underwent radiographic varicocele embolization and was concurrently started on clomiphene citrate. He subsequently had an increase in TMSC to 21.7 ×10^6^ (ejaculate volume 2 mL, sperm concentration 16.4 ×10^6^/mL, motility 66%). Interestingly, electron microscopy performed on the second semen sample, which had a motility of 24%, demonstrated that 70% of the sperm had a loss of the 9-doublet architecture. This man fathered a spontaneous pregnancy after his semen parameters improved after varicocele repair and clomiphene citrate.

Two men underwent electron microscopy for asthenospermia - the one described above (i.e. Patient 4) and one other. Both men had similar results, which demonstrated loss of the characteristic nine doublet microtubule architecture.

## Discussion

Of human chromosomes, the Y chromosome is the only one that is not vital for life [5]. As a result, Y chromosome abnormalities, including deletions, duplications, and inversions are relatively common and not life-threatening [2]. Some of these variants are selectively neutral, and others may cause impaired fertility. The *AZF* region encodes for 21 genes, and its three subregions *AZFa, AZFb,* and *AZFc*, have been mapped to Yq [4]. Today, microdeletions in the *AZF* region are considered one of the primary genetic causes of spermatogenic failure [7]. Nearly all *AZF* microdeletions occur de novo, but once present, they are passed on to all male offspring of an affected man [19].

Among the three *AZF* regions, microdeletions occur most commonly in the *AZFc* region, with de novo deletions arising in roughly 1 in 4000 males [6]. *AZFc* microdeletions are identified in approximately 10% of cases of nonobstructive azoospermia and 5% of cases of severe oligospermia [20]. With the knowledge of the complete Y chromosome sequence, partial microdeletions within the *AZFc* region are now thought to be much more common than complete microdeletions or microduplications [8, 9]. While the complete AZFc microdeletion is well associated with spermatogenic failure, the role of partial *AZFc* deletions on spermatogenesis is still debated [10]. In addition, the reason that these duplications occur exclusively in the *AZFc* region, and not in the *AZFa* or *AZFb* regions, is unknown.

The effects of other *AZFc* rearrangements on male fertility remain unclear, primarily because they are harder to detect or rare. With advances in detection methodology, increasing cases of Yq duplications, especially those involving the *AZFc* region, have been reported [11-15]. In particular, our recently reported custom high-resolution Y-chromosome microarray [16], has enabled us to identify a group of men with complete *AZFc* Y chromosome microduplications. Most of the prior published literature detected Y chromosomal microdeletions by PCR of STS markers that mapped within specific *AZF* regions of the Y chromosome [21]. It is possible that other Y chromosome copy number variations such as duplications and novel deletions may exist which are currently undetectable using routine PCR. Our high-resolution Y chromosome microarray interrogates thousands of different loci on the Y chromosome to detect known Y chromosome microdeletions and uncover other copy number variations of the Y chromosome. It is a direct result of this new technology [17], allowing for more thorough detection of Y chromosomal abnormalities, that allows us to report the first series of men with complete *AZFc* microduplications.

There have been reports of partial *AZFc* microduplications in subfertile males in the Chinese Han population [15] and Chinese Yi population [22], although *AZFc* partial microduplications were not found to impair male fertility in the Italian population [14]. Likewise, a higher incidence of increased number of deleted in azoospermia (*DAZ*) genes has been demonstrated in azoospermic and oligospermic men in Slovenia [16]. Interestingly, two cases of incidentally detected complete Y chromosomal microduplication have been reported. Their fluorescence in situ hybridization (FISH) analysis revealed direct duplication of large segments of short and long arms of the Y chromosome, resulting in nearly two intact Y chromosomes. These men had normal phenotypes and fathered spontaneous pregnancies, although formal semen analyses were not performed [11]. Finally, there have been reports of *AZFa* regional duplications in men from the Iberian Peninsula, although the fertility status of these men was not reported [23]. The men that we identified with complete *AZFc* microduplications were an ethnically diverse population, suggesting that the prevalence of complete *AZFc* microduplications is not ethnically skewed.

It is interesting that while *AZFc* microdeletions are typically considered as de novo genetic abnormalities, the men that we identified as having *AZFc* microduplications did demonstrate a relatively high rate of family history of male factor infertility. Two of these men had fathers who reported a delay in conception, and of those one was also found to have a complete *AZFc* microduplication. In addition, one of the men had a brother who also had a history of oligospermia. While these *AZFc* copy number abnormalities are believed to be copy number dependent, it seems that is a familial predisposition for abnormalities in this region. It is interesting that all of the offspring derived from men with *AZFc* complete microduplications are males and it is reassuring that all are grossly healthy.

The reason for *AZFc* microduplication, either partial or complete, to result in male infertility is unclear. The excess genetic material may increase the expression of genes contained within those regions, such as *DAZ*, encoding RNA-binding proteins, and *CDY1*. This may then disrupt the expression of downstream genes, which may affect spermatogenesis [24]. Finally, it has recently been hypothesized that genomic duplications in *AZFc *can restore copy numbers and serve as a compensatory factor in men with partial *AZFc *microdeletions [12]. Supporting this theory, it has been reported that duplications secondary to partial *AZFc *microdeletions can restore the total motile sperm count to a normal value [25]. Similar to the prior literature, we found heterogeneity of sperm counts in men with complete *AZFc *duplications. One man was persistently azoospermic, the second alternated between severe oligospermia and rare non-motile sperm, the third alternated between azoospermia and severe oligospermia, the fourth was persistently oligospermic, and the fifth man had severe oligospermia, but increased to normospermic counts after intervention.

Likewise, these men had a variety of hormonal levels with FSH ranging from 1-11 IU/L, LH from 3-4.2 IU/L, and testosterone from 12.5-18.3 nmol/L. It is reassuring that in all five men with complete duplications endogenous testosterone production was maintained. The variability in gonadotropin levels likely reflects the individual men’s spermatogenic potential. In general, the gonadotropin levels did correlate with sperm counts.

In this study, we found that complete *AZFc *microduplications represented a relatively high proportion of *AZFc* abnormalities, 21% (5/24 *AZFc* abnormalities). Given that the widely used multiplex PCR is not capable of detecting these *AZFc* microduplications, the incidence of these abnormalities in the general fertile male population is yet unknown, but our prior study on 148 fertile men did not identify any *AZFc* microduplications. The recently developed quantitative fluorescent polymerase chain reaction (QF-PCR) kit is capable of detecting complete *AZFc* microduplications, although this is not yet widely used [17].

While broad conclusions are difficult to draw, it is reassuring that in spite of their genetic abnormality, Patient 4 who underwent intervention (varicocele embolization and medical therapy with clomiphene citrate) had an increase in his TMSC from 1.8 ×10^6^ to 21.7 ×10^6^. After the intervention, he then went on to father a spontaneous pregnancy. The debate on whether interventions in men with genetic causes for spermatogenic failure are warranted due to their intrinsic genetic defect is clearly interesting, but these data suggest that at least some of these men will have a favorable response to interventions. From another perspective, these data are concerning that we may be propagating a population of men with genetic abnormalities.

Limitations of this study include the relatively small number of men actually having *AZFc* microduplications, making meaningful conclusions regarding the effect of duplications on spermatogenesis limited. However, even though this only represents a series of five men, we believe that there is important information to be gained. This reinforces the need for large-scale studies using Y chromosome-specific microarray technology to detect copy number variants, on well-characterized normospermic, oligospermic, and azoospermic individuals of differing ethnic origins so that more patients with this condition can be studied. It is reassuring that three male offspring born to men having *AZFc* microduplications all appear to be grossly healthy, although since they are infants, their fertility status is known. Future studies will focus on further developing these family trees and testing offspring for their *AZFc* status. In addition, we plan to look for individual gene expression in these men, to determine if the mechanism of subfertility in these men is due to a gene dosage effect.

## Conclusions

This is the first study to report that complete *AZFc* microduplications are common in those infertile men with spermatogenic failure, representing 21% of the *AZFc* alterations in this group. We demonstrate demographic and reproductive charactersitics of five men found to have complete *AZFc* microduplications. We also identify a patient with a complete *AZFc* microduplication who responded well to medical and surgical therapy and progressed to father a spontaneous pregnancy. Studies only targeting *AZFc* microdeletions are likely underestimating the frequency of* AZFc *abnormalities in men with spermatogenic failure. Integration of our novel microarray to more accurately characterize AZFc genetic abnormalities may potentially capture additional men with azoospermia or severe oligospermia who would benefit from genetic counseling prior to assisted reproductive technology.
